# A B C of the Swedish System of Educational Gymnastics

**Published:** 1892-03

**Authors:** 


### BOOK NOTICES.

A B C of The Swedish System of Educational Gymnastics. By Hartvig Nissen, Instructor of Physical Training in the Public Schools of Boston, Mass. Instructor of Swedish and German Gymnastics at Harvard University's Summer School, 1891. Author of *A Manual on Swedish Movement and Massage Treatment*, etc. A practical hand-book for school-teachers and the home. Price in United States and Canada, postpaid, 75 cents, net; Great Britain, 4*s.* 6*d.*; France, 4 fr. F. A. Davis, Publisher, Main Office, 1231 Filbert Street, Philadelphia.

This excellent little work is a practical hand-book for teaching the "Swedish Educational Gymnastics." It is true there are some treatises on this subject, but they are not sufficiently practical to be of use as a hand-book. The author of this work has avoided the use of difficult scientific terms and made it as popular and plain as possible.

The first two chapters contain questions and answers such as have been most frequently put to the author in his nearly fourteen years' experience as a teacher of Gymnastics, and these two chapters will give a very satisfactory idea of the foundation of the "Swedish System of Gymnastics." Other chapters contain what are termed prescriptions for daily lessons arranged somewhat as follows:

Five daily orders of exercises for second and third class of primary schools. Six daily orders for the first class of the primary schools. Seven daily orders for the fifth and sixth class of the grammar schools. Nine daily orders for the third and fourth class, and fifteen for the first and second class of the grammar schools.

The fullest instructions and commands are given for each exercise and *seventy-seven excellent engravings* illustrate them and add greatly to the practical value of the book. It is complete in one neat, small 12mo volume of about one hundred and twenty-five pages, and may be conveniently carried in the pocket. Bound in extra flexible cloth.

